# Characteristics of eye disorders induced by atypical antipsychotics: a real-world study from 2016 to 2022 based on Food and Drug Administration Adverse Event Reporting System

**DOI:** 10.3389/fpsyt.2024.1322939

**Published:** 2024-08-02

**Authors:** Chao Mu, Li Chen

**Affiliations:** ^1^ Department of Psychiatry, The Clinical Hospital of Chengdu Brain Science Institute, MOE Key Lab for Neuroinformation, University of Electronic Science and Technology of China, Chengdu, China; ^2^ Department of Pharmacy, West China Second University Hospital, Sichuan University, Chengdu, Sichuan, China

**Keywords:** Food and Drug Administration Adverse Event Reporting System (FAERS), real-world study, atypical antipsychotics, eye disorders, ocular adverse events

## Abstract

**Background:**

Common atypical antipsychotics include risperidone, paliperidone, olanzapine, lurasidone, quetiapine, clozapine, aripiprazole, ziprasidone, asenapine, brexpiprazole, and cariprazine. Previous studies on ocular adverse reactions of antipsychotics were mainly focused on typical antipsychotics. Systematic research on atypical antipsychotics remains limited.

**Objective:**

This study aimed to evaluate the potential risks of different atypical antipsychotics causing ocular side effects by mining the Food and Drug Administration Adverse Event Reporting System (FAERS) database.

**Methods:**

Extract reports from the FAERS from the first quarter of 2016 to the fourth quarter of 2022 were obtained. Data mining of eye disorders associated with atypical antipsychotics was carried out using The Reporting Odds Ratio (ROR) method and The Medicines and Healthcare Products Regulatory Agency (MHRA) method to determine positive signals.

**Results:**

FAERS reports for 9913783 cases were included in these 28 quarters. 64 defined ocular adverse events were classified into 10 categories according to High-Level Group Terms (HLGT).

**Conclusions:**

There were differences in the types and severity of ocular-related adverse events associated with atypical antipsychotics. Ocular neuromuscular-related adverse events were found among all 11 atypical antipsychotics. Olanzapine had the highest signal intensity in oculogyric crisis. Aripiprazole had the highest signal strength in blepharospasm. Cariprazine was associated with cataract-related ocular adverse reactions. In terms of the types of adverse events, our study found that aripiprazole was associated with 28 types of ocular adverse events, followed by quetiapine. Clozapine was only associated with two types of ocular adverse events.

## 1 Introduction

Schizophrenia is a chronic and disabling psychiatric disorder (1). Atypical antipsychotics play important roles throughout treatment. Common Atypical antipsychotics include risperidone (RIS), paliperidone (PAL), olanzapine (OLA), lurasidone (LUR), quetiapine (QUE), clozapine (CLZ), aripiprazole (ARI), ziprasidone (ZIP), asenapine (ASE), brexpiprazole (BRE), and cariprazine (CAR). Compared to typical antipsychotics, atypical antipsychotics have advantages in improving negative symptoms and cognitive function (2).

However, the adverse reactions of atypical antipsychotics should not be overlooked. On one hand, some atypical antipsychotics may induce physical illnesses or syndromes including *diabetes (* 3), QT interval (QTc) prolongation (4), sexual dysfunction (5). On the other hand, patients may discontinue treatment due to adverse reactions, resulting in repeated relapses and ultimately leading to disability (6). Previous studies on ocular toxicity focused mainly on typical antipsychotics (7, 8), but the ocular adverse reactions associated with atypical antipsychotics also warrant attention. While some ocular adverse reactions induced by atypical antipsychotics may be rare (9), failure to detect and treat drug-induced cataracts or glaucoma promptly can lead to irreversible visual impairment or even blindness. Moreover, the majority of reported ocular adverse reactions to atypical antipsychotics, such as ocular myasthenia, central retinal vein occlusion, eye spasms, myopia, diplopia, and macular degeneration, have predominantly been documented through case reports. The relationship and risks associated with these reactions and antipsychotics remain unclear.

Data mining of extensive databases from spontaneous adverse event reporting system has emerged as a crucial method for monitoring drug safety (10). The Food and Drug Administration Adverse Event Reporting System (FAERS), being the largest publicly accessible database worldwide,encompasses millions of adverse drug events reports submitted spontaneously by healthcare professionals, consumers, manufacturers, and others. Our study aims to assess the potential risks of ocular side effects caused by different atypical antipsychotics by analyzing real-world adverse drug events reported in the FAERS database.

## 2 Method

### 2.1 Data source

This study extracted data on adverse drug events from the FAERS database, spanning 28 quarters from the first quarter of 2016 to the fourth quarter of 2022, which included a total of 9,913,783 reports. Healthcare professionals, including physicians, pharmacists, nurses, and others, along with consumers such as patients, family members, lawyers, and manufacturers contribute reports to the FAERS database. The dataset is comprised of seven components: Demographic Characteristics (DEMO), Adverse Event Reactions (REAC), Drugs (DRUG), Adverse Event Results (OUTC), Adverse Event Sources (PRSP), Treatment Times (THER), and Indications (INDI). We imported these seven components into the Relational Database Management System (MySQL) for organization and analysis.

### 2.2 Data screening

Our research investigated atypical antipsychotics, including aripiprazole (tradenames: Abilify^®^, Aristada^®^, etc), asenapine (tradenames: Secuado^®^, Saphris^®^, etc), brexpiprazole (tradename: Rexulti^®^), cariprazine (tradename: Vraylar^®^), clozapine (tradenames: Clozaril^®^, Fazaclo^®^, Versacloz^®^, etc), lurasidone (tradename: Latuda^®^), olanzapine (tradenames: Zyprexa^®^, Lybalvi^®^, etc), paliperidone (tradenames: Invega^®^, Invega Sustenna^®^, etc), quetiapine (tradename: Seroquel^®^, etc), risperidone (tradenames: Risperdal^®^, Risperdal Consta^®^, Rykindo^®^, etc), and ziprasidone (tradename: Geodon^®^). Utilizing drug names sourced from the United States of America Food and Drug Administration (FDA) authorized public dashboard for drugs and adverse reactions, we performed an ambiguous matching of both brand and generic names within the “drug name” field of MySQL database.

Each adverse drug report typically identifies a primary suspect drug (PS) associated with one or more adverse events and may detail additional medications used by the patient. Manufacturers, upon receiving reports from healthcare professionals or consumers, are mandated to relay this information to the FDA according to specific regulations. Due to this process, certain reports may be submitted to the FDA several times with revised details. To maintain data integrity, duplicate reports were identified by case number (ID) and removed, ensuring the inclusion of only the latest submission in the analysis. Additionally, the process of eliminating duplicate reports included verifying matches based on age, sex, event date, and reporter’s country of origin. Ultimately, we evaluated atypical antipsychotics coded as PS following the removal of deduplicates.

### 2.3 Data processing

Adverse events in the FAERS database were coded by Preferred Terms (PTs) in the Medical Dictionary for Regulatory Activities (MedDRA) terminology. In our research, the 64 defined ocular adverse events were classified into 10 distinct categories based on High-Level Group Terms (HLGT) found in MedDRA. These categories include anterior eye structural changes, deposits and degeneration, Other Eye Disorders (NEC), glaucoma and ocular hypertension, ocular hemorrhages and vascular disorders, ocular infections, irritations and inflammations, ocular injuries, ocular neuromuscular disorders, ocular structural changes, deposits and degeneration, retina, choroid and vitreous hemorrhages and vascular disorders, and vision disorders.

### 2.4 Data analysis

Data analysis in our study was conducted using Microsoft Excel 2016. The Reporting Odds Ratio (ROR) method and the Medicines and Healthcare Products Regulatory Agency (MHRA) method were used to calculate RORs and Proportional Reporting Ratios (PRRs), respectively, along with chi-square (χ2) values, to screen for potential adverse drug events signals for atypical antipsychotics. Adverse events with a lower limit of the 95% Confidence Interval (95%CI) greater than 1 and reported in at least three cases using the ROR method, and with PRR > 2, χ2 > 4, and reported in at least three cases using the MHRA method, were defined as adverse drug events signals (**Table 1**). Higher RORs and lower limits of 95% CIs, as well as higher PRRs and χ2 values, suggest stronger associations.

**Table 1:** The calculation formula and threshold of The ROR method and the MHRA method.

Method	Calculation formula	Threshold
ROR method	ROR=(a/c)/(b/d)	When a≥3 and the lower 95%CI of the ROR is greater than 1, 1 ADE signal is generated.
	$\text{ROR}95\text{\%Cl}=\text{exp }\left[1\text{n}\left(\text{ROR}\right)\;\pm \;1.96\sqrt{\frac{1}{\text{a}}+\frac{1}{\text{b}}+\frac{1}{\text{c}}+\frac{1}{\text{d}}}\right]$	
MHRA method	PRR=[a/(a+b)]/[c/(c+d)]	When a≥3, PRR>2 and χ2>4, 1 ADE signal is generated.
	${\text{x}}^{2}=\frac{{\left(\text{ad}-\text{bc}\right)}^{2}\left(\text{a}+\text{b}+\text{c}+\text{d}\right)}{\left(\text{a}+\text{b}\right)\times \left(\text{c}+\text{d}\right)\times \left(\text{a}+\text{c}\right)\times \left(\text{b}+\text{d}\right)}$	

ROR, The Reporting Odds Ratio; MHRA, the Medicines and Healthcare Products Regulatory Agency; PRR, Proportional Reporting Ratios; χ2, chi-square.

CI, Confidence Interval; ADE, Adverse Drug Event; a, a is the number of target ADE of the target drug; b, b is the number of non-target ADE of the target drug.

c, c is the number of target ADE of the non-target drug; d, d is the number of non-target ADE of the non-target drug.

## 3 Results

### 3.1 Ocular neuromuscular disorders adverse events in individual atypical antipsychotics

All studied atypical antipsychotics showed varying degrees of positive signals for ocular neuromuscular disorder adverse events. For Oculogyric Crisis (OGC), strong positive signals were observed with OLA, ARI, QUE, and RIS. Relatively strong positive signals for blepharospasm were seen with ARI, LUR, and QUE. Strong positive signals for miosis were found with OLA and QUE (**Table 2**).

**Table 2:** Ocular neuromuscular disorders-related adverse events of atypical antipsychotics.

PTs	ARI			ASE			CAR			CLZ			BRE			LUR			OLA			PAL			QUE			RIS			ZIP		
	N	PRR	χ2	N	PRR	χ2	N	PRR	χ2	N	PRR	χ2	N	PRR	χ2	N	PRR	χ2	N	PRR	χ2	N	PRR	χ2	N	PRR	χ2	N	PRR	χ2	N	PRR	χ2
**Blepharospasm**	54	7.27	284.71	–	–	–	10	13.83	118.45	–	–	–	14	7.89	83.75	29	11.04	261.23	21	5.93	85.31	10	2.87	12.11	39	7.64	220.91	–	–	–	5	16.25	71.42
**Excessive eye blinking**	19	10.12	150.72	–	–	–	–	–	–	–	–	–	–	–	–	14	21.14	261.68	8	8.90	55.26	7	7.93	41.84	16	12.42	163.00	7	3.01	9.27	–	–	–
**Extraocular muscle disorder**	–	–	–	–	–	–	–	–	–	–	–	–	–	–	–	–	–	–	–	–	–	–	–	–	13	47.89	534.31	–	–	–	–	–	–
**Extraocular muscle paresis**	6	6.47	27.10	–	–	–	–	–	–	–	–	–	–	–	–	–	–	–	8	18.52	128.62	3	6.95	15.11	–	–	–	–	–	–	–	–	–
**Eye movement disorder**	80	8.02	478.06	3	9.06	21.50	9	9.23	65.83	29	2.23	19.41	25	10.49	212.86	24	6.75	116.59	43	9.07	304.22	44	9.47	328.43	30	4.33	76.01	46	3.76	91.74	4	9.65	30.97
**Gaze palsy**	67	27.71	1569.11	–	–	–	–	–	–	–	–	–	–	–	–	–	–	–	23	19.04	380.87	4	3.29	6.34	8	4.46	21.22	18	5.73	68.54	–	–	–
**Miosis**	28	2.34	21.25	–	–	–	–	–	–	–	–	–	–	–	–	–	–	–	144	26.55	3389.99	–	–	–	140	17.71	2116.87	59	4.10	136.06	–	–	–
**Mydriasis**	58	3.12	82.42	–	–	–	–	–	–	–	–	–	–	–	–	–	–	–	74	8.50	483.02	–	–	–	132	10.54	1111.33	–	–	–	–	–	–
**Ocular myasthenia**	–	–	–	–	–	–	–	–	–	–	–	–	–	–	–	–	–	–	4	17.50	60.46	–	–	–	–	–	–	–	–	–	–	–	–
**Oculogyric crisis**	116	37.43	3627.24	4	35.38	133.11	10	30.14	278.92	43	9.98	332.23	25	31.16	711.19	22	18.32	352.25	93	62.32	5079.90	23	14.58	284.04	87	39.80	2998.87	72	18.25	1087.71	9	63.87	551.91
**Ophthalmoplegia**	8	3.56	14.51	–	–	–	–	–	–	–	–	–	–	–	–	–	–	–	–	–	–	–	–	–	–	–	–	–	–	–	–	–	–
**Pupil fixed**	12	7.16	61.98	–	–	–	–	–	–	–	–	–	–	–	–	–	–	–	3	3.74	5.99	–	–	–	–	–	–	–	–	–	–	–	–
**Pupillary reflex impaired**	–	–	–	–	–	–	–	–	–	–	–	–	–	–	–	–	–	–	11	12.96	118.85	–	–	–	–	–	–	8	3.63	14.99	–	–	–
**Pupils unequal**	–	–	–	–	–	–	–	–	–	–	–	–	–	–	–	–	–	–	18	20.52	323.11	–	–	–	–	–	–	–	–	–	–	–	–
**Saccadic eye movement**	5	16.04	66.69	–	–	–	–	–	–	–	–	–	–	–	–	–	–	–	7	49.01	304.14	–	–	–	–	–	–	–	–	–	–	–	–
**Strabismus**	10	2.31	7.39	–	–	–	–	–	–	–	–	–	–	–	–	5	3.29	7.96	–	–	–	–	–	–	–	–	–	–	–	–	–	–	–

RIS, Risperidone; PAL, Paliperidone; OLA, Olanzapine; LUR, Lurasidone; QUE, Quetiapine; CLZ, Clozapine; ARI, Aripiprazole; ZIP, Ziprasidone; ASE, Asenapine; BRE, Brexpiprazole; CAR, Cariprazine; PTs, Preferred Terms; N, Number of reports; PRR, Proportional Reporting Ratio; χ2, Chi-square; –, not a positive signal.

### 3.2 Vision disorders adverse events in individual atypical antipsychotics

Compared with other atypical antipsychotics, we observed more positive signals for vision disorder adverse events with ARI, QUE, CAR, and OLA. A strong positive signal for dysmetropsia was observed with QUE. Only one positive signal each emerged in this category for ASE and RIS (**Table 3**).

**Table 3:** Vision disorders-related adverse events of atypical antipsychotics.

PTs	ARI			ASE			CAR			OLA			QUE			RIS		
	N	PRR	χ2	N	PRR	χ2	N	PRR	χ2	N	PRR	χ2	N	PRR	χ2	N	PRR	χ2
**Accommodation disorder**	15	18.53	233.23	–	–	–	5	62.01	293.91	–	–	–	–	–	–	–	–	–
**Acute myopia**	7	25.38	150.25	–	–	–	–	–	–	–	–	–	–	–	–	–	–	–
**Altered visual depth perception**	–	–	–	–	–	–	–	–	–	–	–	–	6	8.10	36.60	–	–	–
**Amaurosis**	–	–	–	–	–	–	–	–	–	4	7.06	20.58	–	–	–	–	–	–
**Blindness transient**	–	–	–	–	–	–	4	3.57	7.40	–	–	–	–	–	–	–	–	–
**Diplopia**	–	–	–	5	4.15	11.97	–	–	–	–	–	–	–	–	–	–	–	–
**Dyschromatopsia**	–	–	–	–	–	–	4	20.26	72.72	–	–	–	–	–	–	–	–	–
**Dysmetropsia**	–	–	–	–	–	–	–	–	–	–	–	–	8	121.16	735.47	–	–	–
**Glare**	–	–	–	–	–	–	–	–	–	7	19.37	118.14	–	–	–	–	–	–
**Metamorphopsia**	–	–	–	–	–	–	–	–	–	–	–	–	9	4.74	26.28	–	–	–
**Myopia**	16	4.39	41.26	–	–	–	–	–	–	–	–	–	–	–	–	–	–	–
**Night blindness**	10	3.45	17.19	–	–	–	–	–	–	–	–	–	–	–	–	–	–	–
**Optic neuropathy**	–	–	–	–	–	–	–	–	–	–	–	–	12	5.51	43.75	–	–	–
**Photopsia**	21	2.33	15.73	–	–	–	–	–	–	–	–	–	–	–	–	–	–	–
**Presbyopia**	6	8.54	38.78	–	–	–	–	–	–	–	–	–	–	–	–	–	–	–
**Pseudomyopia**	10	52.67	426.40	–	–	–	–	–	–	–	–	–	–	–	–	–	–	–
**Vision blurred**	–	–	–	–	–	–	65	3.21	99.42	–	–	–	–	–	–	–	–	–
**Xanthopsia**	–	–	–	–	–	–	–	–	–	–	–	–	–	–	–	4	7.62	22.25

ARI, Aripiprazole; ASE, Asenapine; CAR, Cariprazine; OLA, Olanzapine; QUE, Quetiapine; RIS, Risperidone; PTs, Preferred Terms; N, Number of reports; PRR, Proportional Reporting Ratio; χ2, Chi-square; –, not a positive signal.

### 3.3 Anterior eye structural change, deposit, and degeneration adverse events in individual atypical antipsychotics

CAR showed significant positive signals for anterior eye structural changes, especially in cataracts. ARI, QUE, and RIS had positive signals for keratoconus. No positive signals were observed for the other atypical antipsychotics (**Table 4**).

**Table 4:** Anterior eye structural change, deposit and degeneration; other ocular structural change, deposit and degeneration: related adverse events of atypical antipsychotics.

HLGT	PTs	ARI			CAR			QUE			RIS		
		N	PRR	χ2	N	PRR	χ2	N	PRR	χ2	N	PRR	χ2
**Anterior eye structural change, deposit and degeneration**	Cataract	–	–	–	23	2.44	19.65	–	–	–	–	–	–
	Cataract cortical	–	–	–	5	261.33	1190.38	–	–	–	–	–	–
	Cataract nuclear	–	–	–	16	224.07	3300.55	–	–	–	–	–	–
	Cataract subcapsular	–	–	–	–	–	–	–	–	–	4	3.19	5.93
	Keratoconus	5	8.36	31.44	–	–	–	4	9.74	30.63	7	9.77	52.89
	Lenticular opacities	–	–	–	4	47.02	177.31	–	–	–	–	–	–
	Xerophthalmia	7	7.66	39.47	–	–	–	–	–	–	–	–	–
**Other ocular structural change, deposit and degeneration**	Chorioretinopathy	–	–	–	–	–	–	12	5.63	45.12	–	–	–
	Lid sulcus deepened	3	4.27	7.41	–	–	–	–	–	–	–	–	–
	Optic discs blurred	–	–	–	–	–	–	9	147.22	961.03	–	–	–
	Papilloedema	–	–	–	–	–	–	13	2.73	14.16	–	–	–
	Retinal degeneration	7	3.73	13.80	–	–	–	–	–	–	–	–	–
	Scleral discolouration	–	–	–	–	–	–	10	14.40	120.45	–	–	–

HLGT, High-Level Group Terms; ARI, Aripiprazole; CAR, Cariprazine; QUE, Quetiapine; RIS, Risperidone; PTs, Preferred Terms; N, Number of reports; PRR, Proportional Reporting Ratio; χ2, Chi-square; –, not a positive signal.

### 3.4 Ocular structural change, deposit, and degeneration adverse events in individual atypical antipsychotics

Positive signals for ocular structural changes, deposits, and degeneration were mainly concentrated in QUE, which had a strong positive signal for optic discs blurred. Several positive signals also emerged in this category for ARI. No positive signals were observed for the other atypical antipsychotics (**Table 4**).

### 3.5 Eye disorders NEC adverse events in individual atypical antipsychotics

In this part, we only observed positive signals in OLA, PAL, and ZIP. Periorbital edema and swelling were associated with OLA. Lacrimation decreased was associated with PAL. And ocular discomfort was associated with ZIP. (**Table 5**).

**Table 5:** Other Eye disorders; Glaucoma; Ocular Haemorrhages and Vascular Disorders; Ocular Infections, Irritations and Inflammations; Ocular Injuries; Retina, Choroid and Vitreous Haemorrhages and Vascular Disorders: Related Adverse Events of Atypical Antipsychotics.

HLGT	PTs	ARI			ASE			OLA			PAL			QUE			RIS			ZIP		
		N	PRR	χ2	N	PRR	χ2	N	PRR	χ2	N	PRR	χ2	N	PRR	χ2	N	PRR	χ2	N	PRR	χ2
**Other Eye disorders**	Lacrimation decreased	–	–	–	–	–	–	–	–	–	3	8.51	19.61	–	–	–	–	–	–	–	–	–
	Ocular discomfort	–	–	–	–	–	–	–	–	–	–	–	–	–	–	–	–	–	–	3	3.81	6.22
	Periorbital oedema	–	–	–	–	–	–	12	3.41	20.28	–	–	–	–	–	–	–	–	–	–	–	–
	Periorbital swelling	–	–	–	–	–	–	9	2.12	5.34	–	–	–	–	–	–	–	–	–	–	–	–
**Glaucoma**	Angle closure glaucoma	12	3.18	17.78	–	–	–	20	11.40	186.20	–	–	–	24	9.44	176.96	–	–	–	–	–	–
**Ocular haemorrhages and vascular disorders**	Eyelid bleeding	–	–	–	–	–	–	3	23.18	61.30	–	–	–	–	–	–	–	–	–	–	–	–
	Ophthalmic vein thrombosis	4	6.61	18.59	–	–	–	–	–	–	–	–	–	–	–	–	–	–	–	–	–	–
**Ocular infections, irritations and inflammations**	Birdshot chorioretinopathy	5	43.61	180.05	–	–	–	–	–	–	–	–	–	–	–	–	–	–	–	–	–	–
	Choroiditis	5	7.05	25.31	–	–	–	–	–	–	–	–	–	–	–	–	–	–	–	–	–	–
	Cystoid macular oedema	–	–	–	–	–	–	–	–	–	–	–	–	–	–	–	19	4.89	57.49	–	–	–
	Eyelid oedema	–	–	–	3	5.82	11.99	–	–	–	–	–	–	–	–	–	–	–	–	–	–	–
	Keratitis	–	–	–	–	–	–	12	5.40	42.66	–	–	–	–	–	–	–	–	–	–	–	–
	Orbital myositis	–	–	–	–	–	–	–	–	–	–	–	–	–	–	–	4	9.60	29.58	–	–	–
**Ocular injuries**	Ocular toxicity	–	–	–	–	–	–	5	7.00	25.42	–	–	–	–	–	–	–	–	–	–	–	–
**Retina, choroid and vitreous haemorrhages and vascular disorders**	Retinal exudates	–	–	–	–	–	–	–	–	–	–	–	–	3	3.93	6.50	6	4.47	15.87	–	–	–
	Retinal vein occlusion	–	–	–	–	–	–	12	6.80	58.71	–	–	–	13	5.07	41.93	10	2.19	6.40	–	–	–
	Retinal white dots syndrome	3	167.48	310.28	–	–	–	–	–	–	–	–	–	–	–	–	–	–	–	–	–	–

HLGT, High-Level Group Terms; ARI, Aripiprazole; ASE, Asenapine; OLA, Olanzapine; PAL, Paliperidone; QUE, Quetiapine; RIS, Risperidone; ZIP, Ziprasidone; PTs, Preferred Terms; N, Number of reports; PRR, Proportional Reporting Ratio; χ2, Chi-square; –, not a positive signal

### 3.6 Glaucoma adverse events in individual atypical antipsychotics

3 Atypical antipsychotics showed positive signals for glaucoma - ARI, OLA, and QUE. OLA and QUE exhibited higher signal strength compared to ARI (**Table 5**).

### 3.7 Ocular hemorrhages and vascular disorders adverse events in individual atypical antipsychotics

In this category, we only observed positive signals of ARI and OLA, which were ophthalmic vein thrombosis and eyelid bleeding respectively. (**Table 5**).

### 3.8 Ocular infections, irritations, and inflammations adverse events in individual atypical antipsychotics

We observed positive signals of ARI, OLA, ASE, and RIS in this aspect. Notably, a relatively strong positive signal was observed with birdshot chorioretinopathy in ARI. (**Table 5**).

### 3.9 Ocular injuries adverse events in individual atypical antipsychotics

In this part, we only observed that ocular toxicity was associated with the use of OLA. No positive signals were observed in the rest of the atypical antipsychotics. (**Table 5**).

### 3.10 Retina, choroid, and vitreous hemorrhages and vascular disorders adverse events in individual atypical antipsychotics

QUE and RIS both showed two identical positive signals - retinal exudates and retinal vein occlusion. One positive signal each was observed for OLA and ARI. Notably, extraordinarily strong positive signals were seen for Retinal white dots syndrome with ARI (**Table 5**).

## 4 Discussion

### 4.1 Olanzapine (OLA)

Our study found that all studied atypical antipsychotics exhibited varying degrees of signals for two adverse reactions – OGC and eye movement disorder. Among them, OLA showed the strongest signal intensity. Previous studies have also reported some OGC cases induced by OLA, with symptoms appearing within days to a week of starting medication. This may be related to higher dopamine-acetylcholine antagonism or inhibition of the dopamine (DA) function of OLA in the striatum (11). Furthermore, OGC with OLA appears dose-dependent, as higher doses have high D_2_ affinity, increasing OGC risk (12). OLA also showed strong positive signals in both closed angle glaucoma and mydriasis. Acute Closed Angle Glaucoma (ACAG) has been reported to be associated with many typical antipsychotics. The mechanism may be due to pupillary blockage. Mydriasis can trigger ACAG through sympathetic stimulation or parasympathetic inhibition. With mild anticholinergic activity, OLA may potentially induce ACAG (13). Blepharospasm is common in Meige syndrome or Brueghel syndrome (14). In a study, eight atypical antipsychotics were identified as having indications of causing blepharospasm. Notably, OLA demonstrated a moderate intensity of this side effect. This finding contrasts with prior studies, which predominantly associated blepharospasm with typical antipsychotics (15). Another research suggested that OLA-induced blepharospasm often appeared after 6 months, which might be related to the long-term use of OLA leading to DA receptor hypersensitivity (16). OLA was also associated with retinal vein occlusion, aligning with prior case reports (17, 18). By blocking platelet 5-Hydroxytryptamine Receptor 2A (5-HT_2A_) receptors, OLA may increase platelet aggregation and vascular contraction, contributing to central retinal vein occlusion (CRVO) (17). Sedative effects, weight gain, and hyperprolactinemia may also impart a prothrombotic state (18). Moreover, we also found a positive signal for periorbital edema, potentially due to OLA’s effects on renal DA and fluid balance (19). Additional signals of varying intensities were observed for pupil fixed, amaurosis, ocular toxicity, keratitis, excessive eye blinking, ocular myasthenia, eyelid bleeding, glare, impaired pupillary reflex, saccadic eye movement, unequal pupils, and gaze palsy. Relevant reports are lacking currently. No cataract signal was observed. The relationship between OLA and cataracts remains controversial, with a study suggesting antipsychotic protective effects (20), while others showed increased cataract risk (21), Poor nutrition in schizophrenia itself raised cataract risk (22). As *diabetes* from OLA adverse effects increases cataract risk fourfold (23), monitoring is still warranted despite rarity (9), No anterior segment pigmentary deposit signal was observed either. It is more commonly associated with Typical antipsychotics such as fluphenazine or chlorpromazine, though one case reported that a patient developed bilateral diffuse corneal pigmentation after taking OLA for 2 years (24). This may be a rare ocular adverse effect of OLA. The mechanism may be like that of Typical antipsychotics, involving drug decomposition induced by ultraviolet light and affinity for 5-Hydroxytryptamine (5-HT)/DA receptors (24).

### 4.2 Aripiprazole (ARI)

ARI had the most ocular adverse reaction positive signals (28 total) among the studied atypical antipsychotics. Broad signals were observed for ocular neuromuscular disorders, especially OGC, gaze palsy, and eye movement disorder. Current studies suggest that OGC is a rare Extrapyramidal Syndrome (EPS) manifestation associated with ARI (25, 26). Based on its pharmacological profile as a partial agonist at Dopamine2 (D2), Dopamine3 (D3), and 5-HT_1A_ receptors, and antagonist at 5-HT_2A_ receptors, ARI modulates dopaminergic activity but lacks anticholinergic effects. This may increase the risk of EPS like OGC (27). In the vision disorders category, positive signals for pseudomyopia and acute myopia were prominent, indicating a strong correlation between ARI and drug-induced myopia. Some prior studies supported this association as well (28, 29). Proposed mechanisms include ARI leading to the ciliary body and choroidal effusion and swelling, causing anterior displacement of ciliary processes, ciliary sulcus narrowing, and forward iris and lens displacement. Alternatively, ARI may directly enter the lens, altering osmosis and causing swelling and myopia (29). This reaction appears reversible with dose reduction or discontinuation. Some studies suggested angle-closure glaucoma as a rare ARI adverse event (30). We also observed that ARI was associated with angle-closure glaucoma. By blocking the iris and ciliary muscle 5-HT/α1 receptors, ARI can cause mydriasis and lens-iris displacement, contributing to ACAG (30). Associations between ARI and choroidal retinal changes were also found, potentially from ARI blocking Dopamine4 (D4) receptors, reducing melatonin synthesis, and increasing the sensitivity of photoreceptors to light-induced damage (31). Additional positive signals requiring further research were observed for conditions like lid sulcus deepened, retinal degeneration, ophthalmic vein thrombosis, choroiditis, keratoconus, xerophthalmia, and retinal white dots syndrome.

### 4.3 Quetiapine (QUE)

Our study found QUE to be associated with various ocular neuromuscular disorders including OGC, miosis, mydriasis, and others. A strong positive signal for OGC was observed with QUE. One case report documented OGC in a patient after taking QUE (32). However, another study suggested that QUE could improve antipsychotic-induced OGC, potentially due to the lower D2 receptor occupancy and 5-HT2 receptor affinity of QUE (33). Given this controversy, further research is warranted to elucidate this relationship. One study reported irreversible visual impairment and branch retinal vein occlusion in a patient after 3 years of QUE treatment, which may be related to abnormal lipid metabolism associated with long-term QUE use (34). We observed a strong positive signal of QUE in the background of ACAG. QUE-induced ACAG may be attributable to the strong anticholinergic effect of its metabolite norquetiapine. Use of QUE in specific populations, such as those taking anticholinergic medications (35) or with Intraoperative Floppy Iris Syndrome (IFIS) (36) may increase ACAG risk. Positive signals were also exhibited for ocular structural changes, deposits, and degeneration like optic disc blurring, scleral discoloration, chorioretinopathy, and papilledema. One case reported decreased left eye vision in a patient taking 200 mg/day QUE, with optical coherence tomography showing chorioretinopathy. Serotonin-mediated ciliary body choroidal effusion has been proposed as a mechanism for QUE-associated transient myopia (37). Our study identified a correlation between QUE and several vision disorders, such as dysmetropsia, optic neuropathy, altered visual depth perception, and metamorphopsia. However, these adverse reactions have not been reported yet and further research is needed to clarify the relationship. For cataracts, no signal was observed, although long-term use of QUE leading to cataracts has been reported (38). The causal relationship between QUE and lens abnormalities remains unclear, as does the cataractogenic potential of QUE (39). However, cataract patients taking QUE may develop IFIS during cataract surgery (36, 40). The mechanism can be attributed to the antagonistic effect of QUE on α1-adrenergic receptors. Compared with dedicated α1-adrenergic receptor antagonists, IFIS induced by QUE is typically milder in presentation (36).

### 4.4 Risperidone (RIS)

A study reported that patients experienced OGC independently of EPS after taking RIS (41). We also observed a strong positive signal for OGC, which may be related to the relatively strong DA receptor-blocking effect of RIS on the nigrostriatal pathway (42). In addition to OGC, RIS was associated with various other ocular neuromuscular disorders. Case reports described patients experiencing blepharospasm, suggested by difficulty eye-opening and improving with medication cessation or switching (43, 44). RIS was also associated with cystoid macular edema, which can arise from medications, surgeries, or other factors and may cause vision loss. The potential mechanism of RIS-induced cystoid macular edema may involve alpha-adrenergic receptor blockade, causing vasodilation or direct retinal endothelial effects (45). The vision loss caused by RIS-induced cystoid macular edema appeared to be benign and reversible (45, 46). We also observed an association between RIS and xanthopsia. One case documented transient yellow vision occurring two days following the initiation of RIS treatment, which resolved upon discontinuation of the medication. This phenomenon could potentially be linked to RIS’s DA-antagonism effects, impacting the functional integrity of the rods and cones in the retina (47). Regarding cataracts, we observed a weak positive signal with only four reported cases. Some studies noted cataract onset from 1–10 years of RIS treatment (48, 49). Therefore, we recommend visual health management for patients on long-term RIS therapy. Additionally, further research is needed to clarify the relationship between cataracts and RIS. Other unreported events like orbital myositis and keratoconus require more research.

### 4.5 Cariprazine (CAR)

CAR was associated with several ocular neuromuscular adverse events, including OGC, blepharospasm, and eye movement disorder. Among the studied atypical antipsychotics, CAR had the weakest signal for OGC, with moderate signals for eye movement disorder and blepharospasm. This suggests CAR may have relatively lower risks of ocular neuromuscular effects versus other atypical antipsychotics. The strongest positive signal (PRR:224.06, χ2:3300.54) was observed for CAR regarding cataract-related adverse events. One animal trial found that dogs developed cataract-related symptoms after 13 weeks of CAR administration (50), although another study showed only approximately 0.1% of patients had cataract-related symptoms after 48 weeks, with the correlation remaining controversial (51). Positive signals were also observed for vision disorders, including accommodation disorder, dyschromatopsia, blurred vision, and transient blindness. Blurred vision (N:65, χ2: 99.42) was the most reported ocular adverse event in CAR. This was consistent with other studies. A study on the efficacy and tolerance of CAR in patients with bipolar disorder suggested that blurred vision was a common adverse effect, and its incidence was similar to that of sedation (52). A pediatric study also found that 10% of patients had CAR-associated blurred vision (53). Given blurred vision is a manifestation of cataracts, longitudinal ocular monitoring of CAR patients appears warranted.

### 4.6 Paliperidone (PAL)

PAL, also known as 9-hydroxyrisperidone, was associated with fewer ocular adverse event types compared to RIS. Currently reported adverse events include OGC during PAL initiation (54) and blepharospasm with long-term use of long-acting paliperidone injections (55). This is consistent with our positive signal findings for OGC and blepharospasm. We also found an association between PAL and decreased Lacrimation.

### 4.7 Lurasidone (LUR)

There is limited research on ocular adverse effects of LUR. Case reports documented OGC after a patient increased the LUR dose to 160 mg/day (56), and blepharospasm after 4 years of treatment with 80 mg/day LUR (57). All observed LUR-associated ocular adverse events involved the ocular neuromuscular system, including OGC, blepharospasm, strabismus, eye movement disorder, and excessive eye blinking.

### 4.8 Ziprasidone (ZIP)

ZIP may lead to OGC. A case report documented OGC and eye movement disorder in a teenage male after low-dose ZIP administration (40mg/day) (58). Additionally, a female schizophrenia patient was diagnosed with OGC secondary to ZIP exposure (59). This aligns with our observations that ZIP-associated adverse events were mainly concentrated in OGC, blepharospasm, and movement disorder. We also observed a positive signal for ocular discomfort with ZIP.

### 4.9 Asenapine (ASE)

For ASE, we observed only four ocular adverse event signals - OGC, eye movement disorder, diplopia, and eyelid oedema. Compared to other atypical antipsychotics, ASE had weaker signal intensity and fewer adverse events. Limited research is available on ASE ocular effects, with only a case report of eyelid edema (60). Therefore, ASE appears relatively safe regarding ocular adverse reactions.

### 4.10 Brexpiprazole (BRE)

There appears to be a lack of research specifically examining the ocular adverse effects of BRE. In our study, BRE was associated with the fewest ocular adverse event types among the studied atypical antipsychotics, apart from CLZ, including oculogyric crisis, eye movement disorder, and blepharospasm. We suggest patients taking BRE should primarily monitor for changes in ocular nerve and muscle function.

### 4.11 Clozapine (CLZ)

Our results demonstrated that CLZ was associated with only two ocular neuromuscular disorders -OGC and eye movement disorder. Limited studies have mentioned OGC as a CLZ adverse effect (61). CLZ is often used to ameliorate OGC, as its high Dopamine1 (D1) receptor blockade and low D2 blockade can prevent D1/D2 imbalance, making OGC less likely (62). However, in addition to its ocular neuromuscular adverse reactions, existing studies suggested potential associations between CLZ and dry eye syndrome (63), periorbital edema (64), macular degeneration (65), blepharospasm (66), blepharal pigmentation (67), cataract (21). CLZ-induced dry eye syndrome may be related to its anticholinergic effect and impairment of the harmony of eye blink reflex (63). Periorbital edema may be due to CLZ blocking renal D4 receptors, preventing them from performing their normal natriuretic and diuretic functions (64). Although CLZ is often used to treat blepharospasm (68, 69). It blockade of DA receptors may lead to receptor hypersensitivity, which is a potential mechanism of leading to blepharospasm (66). In conclusion, CLZ is associated with some ocular neuromuscular adverse events. Other adverse events may be rarer and require further study.

### 4.12 High-risk patients

Our study has discovered that all currently available atypical antipsychotics carry the risk of ocular adverse events, though the severity and number of these events vary among different drugs. Hence, we propose that patients on higher doses of atypical antipsychotics and those with pre-existing ocular diseases about to initiate atypical antipsychotic therapy should be considered at high risk. Such patients should avoid concomitant use of other drugs known for ocular toxicity to minimize the cumulative risk of adverse reactions. For these high-risk individuals, regular examinations including OGC, blepharospasm, cataracts, and glaucoma screenings should be conducted, along with close collaboration with ophthalmologists to manage and monitor the risk of ocular adverse effects.

## 5 Limitations

1. The ROR method and MHRA method can only be used to describe the strength of the relationship between drugs and adverse events but cannot determine causality. More controlled trials and cohort studies may be needed in the future to clarify this.

2. Reports of the FAERS database are submitted spontaneously, which may lead to inaccuracies, false reports, and inevitably introduce bias.

3. Our approach involved prioritizing drugs coded as PS during the selection process, which to some extent, avoided the interference caused by polypharmacy. However, reality is often more complex, encompassing various scenarios such as adverse events caused by PS, those triggered by other medications, or adverse events resulting from the combined use of multiple drugs. In our future research, we plan to extract and analyze data concerning the concomitant use of medications. For instance, we intend to compare the adverse events of using risperidone alone to those where risperidone is PS but used in combination with olanzapine, observing any differences in ocular adverse events. Although this may not yield the outcomes we anticipate, it remains a valuable method worth exploring.

## 6 Conclusions

There were differences in the types and severity of ocular-related adverse events associated with atypical antipsychotics. Ocular neuromuscular-related adverse events were found among all 11 Atypical antipsychotics. Olanzapine had the highest signal intensity in oculogyric crisis. Aripiprazole had the highest signal strength in blepharospasm. Cariprazine was associated with cataract-related ocular adverse reactions. In terms of the types of adverse events, our study found that aripiprazole was associated with 28 types of ocular adverse events, followed by quetiapine. Clozapine was only associated with two types of ocular adverse events. For some ocular adverse events with a small number of cases, further research is still needed to clarify the internal relationship.

## Data availability statement

The datasets presented in this study can be found in online repositories. The names of the repository/repositories and accession number(s) can be found here: https://www.fda.gov/drugs/questions-and-answers-fdas-adverse-event-reporting-system-faers/fda-adverse-event-reporting-system-faers-latest-quarterly-data-files.

## Author contributions

CM: Writing – original draft. LC: Conceptualization, Data curation, Methodology, Writing – review & editing.
